# Characterization of the complete mitochondrial genome of *Chionobathyscus dewitti* (Perciformes, Channichthyidae)

**DOI:** 10.1080/23802359.2019.1688112

**Published:** 2019-11-08

**Authors:** Sapto Andriyono, Md. Jobaidul Alam, Soo Rin Lee, Seok-Gwan Choi, Sangdeok Chung, Hyun-Woo Kim

**Affiliations:** a Interdisciplinary Program of Biomedical, Mechanical and Electrical Engineering, Pukyong National University, Busan, Republic of Korea;; b Fisheries and Marine Faculty, Universitas Airlangga, Surabaya, Indonesia;; c National Institute of Fisheries Science (NIFS), Busan, Republic of Korea;; d Department of Marine Biology, Pukyong National University, Busan, Republic of Korea

**Keywords:** Icefish, next-generation sequencing, *Chionobathyscus dewitti*, Antarctic

## Abstract

The complete mitochondrial genome sequence of the Icefish, *Chionobathyscus dewitti* was determined by the Next Generation Sequencing (NGS) analysis. The complete mitogenome was 17,452 bp in length, which encoded the canonical 13 protein-coding genes, 22 tRNAs, two rRNAs, and two non-coding regions. As shown in the other notothenids, translocation of ND6 and an additional non–coding region were identified, which is different from the typical vertebrate mitochondrial genomes. The *C. dewitti* was clustered distinctly from the those in the *Chinodraco* and *Chaenocephalus*, which supported the idea that this species should be classified in the different genus, *Chionobathyscus* in the family Channichthyidae.

*Chionobathyscus dewitti* is the species in the family Channichthyidae in the Southern Ocean, which is also known as one of the major prey object of Antarctic toothfish, *Dissostichus mawsoni* (Yoon et al. 2017). The blood of fish in the family Channichthyidae is colorless by lacking the functional hemoglobin gene as an evolutional adaptation strategy to the extreme cold water temperature (Ruud 1954). Although it is believed to be related to the formation of the Antarctic Polar Frontal Zone (APFZ) and Antarctic Circumpolar Current (ACC), the evolutional relationship of notothenioid fish is still not fully understood (Kock 2005). In fact, only six mitochondrial genome sequences are currently reported among the 33 currently reported icefish species according to World Register of Marine Species (WoRMS). We here report the complete mitochondrial genome of *C. dewitti,* which was collected from Antarctic Ocean and analyzed its phylogenetic position within the family members.

The specimen was collected from Antarctic subarea 58.4.1(65°13'29.6”S 138°34'21.0”E) as the scientific survey in 2018 and the species identification was confirmed by both the morphological characteristics and the sequence identity (99.85%) in its COI region (HQ712909). The specimen and its DNA are stored at the Marine Biodiversity Institute of Korea (MABIK GR00002617). The complete mitochondrial genome of *C. dewitti* was determined by assembling the raw reads generated by Illumina MiSeq sequencer (Illumina, San Diego, USA). The mitochondrial DNA was extracted with a commercially available kit (Abcam, Cambridge, MA, USA) and a library was constructed by TruSeq^®^ RNA library preparation kit V2 (Illumina, San Diego, USA). Geneious Prime software (Kearse et al. 2012) and tRNAScan-SE program (Lowe and Chan 2016) was used for construction of circular mitochondrial genome and for prediction of the secondary structures of tRNAs. The phylogenetic tree was constructed by MEGA7 with the Minimum Evolution (ME) algorithm (Kumar et al. 2016)

The complete circular mitogenome of *C. dewitti* (MN104591) was 17,452 bp in length, which consisted of 13 protein-coding genes, 22 tRNAs, and two ribosomal RNAs (12S and 16S). The control region was observed between tRNA-*Pro* and tRNA-*Phe*, while the O_L_ was located between tRNA*^Asn^* and tRNA*^Cys^* at the WANCY tRNA cluster similar to other fish species (Andriyono et al. 2018; Dong et al. 2017). Except for the tRNA*^Ser-GCT^*, all the other 21 tRNAs were predicted to be folded into the typical clover-leaf structures. Translocation of ND6 and an additional non–coding region were also identified as shown in those of the other notothenids including *Notothenia coriiceps* (Oh et al. 2016), *Chaenodraco wilsoni* (Dong et al. 2017), and *Chaenocephalus aceratus* (Lee et al. 2015). Unusual start codon (AGG) was identified in ND6 while the incomplete stop codons (TA-/T–) were identified in seven genes, including ND2, COX2, COX3, ND3, ND4, ND5, and CYT B. The phylogenetic tree showed that *C. dewitti* was distinct from the those in the *Chinodraco* and *Chaenocephalus* among the currently reported mitogenomes supporting the idea that this species should be classified in the different genus, *Chionobathyscus* in the family Channichthyidae (Figure 1).

**Figure 1.**
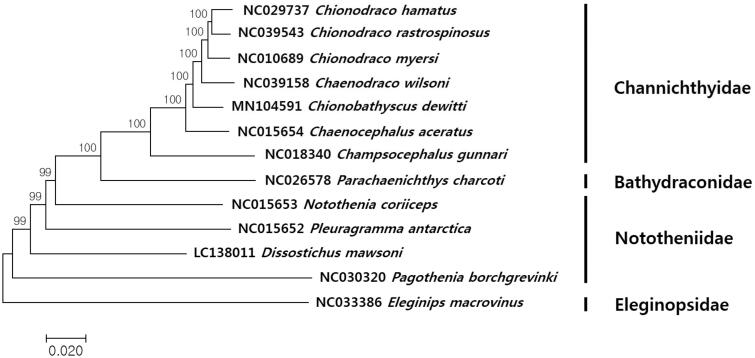
Phylogenetic relationship of *Chionobathyscus dewitti* in the order Perciformes. A phylogenetic tree was constructed with the currently reported complete mitochondrial genome in the order Perciformes by MEGA7 software using Minimum Evolution (ME) algorithm with 1000 bootstrap replications. GenBank accession numbers were shown followed by each species scientific name.
